# Peer Counseling in a Community Based Intervention for Unaccompanied Immigrant Youth

**DOI:** 10.1192/j.eurpsy.2022.151

**Published:** 2022-09-01

**Authors:** A. Qureshi, N. Morales, F. Collazos

**Affiliations:** 1 Hospital Universitari Vall d’Hebron, Psychiatry, Barcelona, Spain; 2 Hospital Universitari Vall d’Hebron, Psychiatry Department, Barcelona, Spain

**Keywords:** transcultural, migration, racism

## Abstract

**Abstract Body:**

Uaccompanied migrant youth represent an at-risk population given the complexity of negotiating adolescence in a new culture, isolated from family and friends, without a secure base and subject to discrimination. In addition, many unaccompanied migrant youth have been subject to considerable trauma prior to, during, and post migration. In Spain, as in many countries, the residential, care, and mental health services are not adapted to the specific and complex needs of this population, and to that end complex not only are the youth not well served but providers are increasingly frustrated. The figure of the community health agent has been widely recognized as one that can function as an effective bridge between systems/institutions and marginalized and vulnerable populations. In this presentation we will describe an ongoing project that trains unaccompanied migrant youth who show promise in their cultural adaptation in the areas of cultural competence, mental health care and substance abuse to function as community health agents (or peer counselors) consistent with models of cultural consultation.

**Disclosure:**

No significant relationships.

